# Neuroprotective Effects of Carnosic Acid: Insight into Its Mechanisms of Action

**DOI:** 10.3390/molecules28052306

**Published:** 2023-03-02

**Authors:** Fatima Javed Mirza, Saadia Zahid, R. M. Damian Holsinger

**Affiliations:** 1 Laboratory of Molecular Neuroscience and Dementia, School of Medical Sciences, Faculty of Medicine and Health, The University of Sydney, Camperdown, NSW 2050, Australia; 2 Neurobiology Research Laboratory, Department of Healthcare Biotechnology, Atta-ur-Rahman School of Applied Biosciences, National University of Sciences and Technology, Islamabad 44000, Pakistan; 3 Neuroscience, School of Medical Sciences, Faculty of Medicine and Health, The University of Sydney, Sydney, NSW 2006, Australia

**Keywords:** neuroprotection, carnosic acid, natural sources, neurodegeneration, autophagy, oxidative stress, apoptosis, Keap1/Nrf2 signaling

## Abstract

Carnosic acid is a diterpenoid abundantly present in plants belonging to the genus *Rosmarinus* and *Salvia* of the family *Lamiaceae,* accounting for their application in traditional medicine. The diverse biological properties of carnosic acid that include antioxidant, anti-inflammatory, and anticarcinogenic activities have instigated studies on its mechanistic role, providing further insights into its potential as a therapeutic agent. Accumulating evidence has established the relevance of carnosic acid as a neuroprotective agent exhibiting therapeutic efficacy in combatting neuronal-injury-induced disorders. The physiological importance of carnosic acid in the mitigation of neurodegenerative disorders is just beginning to be understood. This review summarizes the current data on the mode of action through which carnosic acid exerts its neuroprotective role that may serve to strategize novel therapeutic approaches for these debilitating neurodegenerative disorders.

## 1. Introduction

Neuronal injury is a major factor contributing to various neurological disorders. Despite advancements in the field of medicine and neuroscience, most neurological disorders remain incurable. Currently approved drugs for the treatment of neurological disorders focus on symptomatic relief rather than cure. Recently, there has been an interest in natural products and their therapeutic potential against these disorders. The plants from the genus *Rosmarinus* and *Salvia,* belonging to the family *Lamiaceae,* are the natural sources of carnosic acid (CA) and other natural compounds, which are being widely studied for their therapeutic effects against various conditions [1].

*Salvia Rosmarinus*, belonging to the family Lamiaceae, is native to the Mediterranean but is now found abundantly throughout the world. Commonly referred to as ‘rosemary’, it has been used as an herbal spice in food and has been a constituent of traditional therapies for various illnesses, including inflammatory diseases, headaches, and gastrointestinal issues [2,3]. Rosemary possesses significant intrinsic antioxidant activity that has been attributed to its major constituents, rosmarinic acid and carnosic acid (CA), which have demonstrated neuroprotective activity in various neurodegenerative diseases [4].

**Figure 1:**
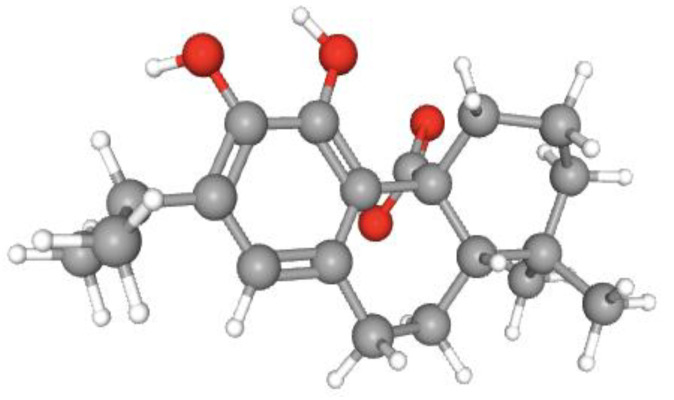
Three-dimensional structure of carnosic acid, acquired from PubChem (pubchem.ncbi.nlm.nih.gov—accessed on 18 January 2023). White represents hydrogen, grey represents carbon and red represents oxygen.

CA is the most abundant compound in rosemary leaves, accounting for 90% of its antioxidant activity [5]. CA is a diterpenoid with an abietane skeleton. Its structure comprises abieta-8,11,13-triene substituted by hydroxy groups at positions 11 and 12 and a carboxy group at position 20 [6]. This carbotricyclic polyphenolic compound is a monocarboxylic acid and a conjugate acid of a carnosate [6]. It is a lipophilic antioxidant that scavenges singlet oxygen, hydroxyl radicals, and lipid peroxyl radicals, thus preventing lipid peroxidation and disruption of biological membranes [7,8] (Figure 1).

CA possesses diverse biological properties, including antioxidant, anti-inflammatory, neuroprotective, and anticarcinogenic activity [8,9,10,11]. However, the mechanisms by which CA exerts its neuroprotective effect have not been fully elucidated, and ongoing studies are providing insight into possible mechanisms of action. In this review, we aim to systematically discuss the potential neuroprotective properties and mechanisms of action of CA to provide a better understanding of its efficacy as a therapeutic agent in neural-injury-associated disorders.

## 2. Carnosic Acid and Mechanisms of Neuroprotection

**Figure 2:**
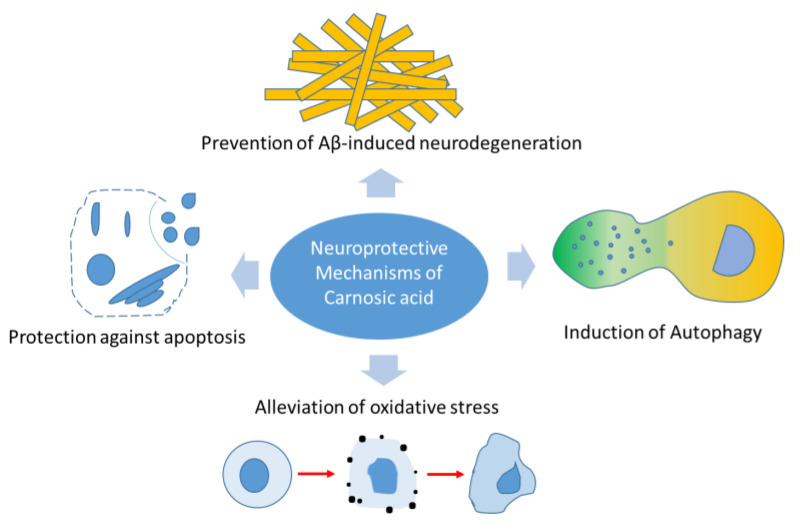
Potential neuroprotective mechanisms of carnosic acid.

CA exerts its neuroprotective effects through a diverse range of mechanisms, some of which include the prevention of amyloid-β (Aβ)-induced neurodegeneration, induction of autophagy, alleviation of oxidative stress and via anti-apoptotic effects (Figure 2). We systematically review these mechanisms to elucidate the potential of CA in the prevention and control of neural-injury-associated disorders.

### 2.1. Induction of Autophagy 

The pathogenesis of most neurodegenerative disorders bears a resemblance to the manner in which the pathogenic proteins are disposed of by neurons and glia. Autophagy, a homeostatic process by which the degradation of long-lived cellular proteins, lipids, and dysfunctional organelles occur within the lysosomal machinery, plays a crucial role in maintaining the metabolic balance between synthesis, degradation, and subsequent turnover of cytoplasmic material [12,13,14]. Since it prevents the buildup of protein aggregates and damaged mitochondria and organelles, loss of autophagy or its dysregulation may lead to atrophy and neuronal death [15]. Autophagic dysregulation is also implicated in neurodegenerative disorders such as Alzheimer’s disease (AD), Parkinson’s disease (PD), Huntington’s disease (HD), amyotrophic lateral sclerosis (ALS), and lysosomal storage disorders (LSDs) [14].

A study employing human neuroblastoma SH-SY5Y cells revealed an instrumental role for CA in the reduction of Aβ-induced apoptosis and the accumulation of toxic proteins through the induction of autophagy. Aβ aggregation is a hallmark feature of AD and is a key target of AD-related therapies. The study by Liu and colleagues demonstrated that CA-induced autophagy via AMP-activated protein kinase (AMPK) is an important regulator of cellular metabolism [16]. AMPK triggers autophagy to avoid oxidative stress and mitochondrial dysfunction in cells treated with CA, highlighting a therapeutic mechanism of CA against Aβ [16]. In vitro studies that investigated the effect of pre-treating SH-SY5Y cells with CA prior to serum starvation revealed that pretreatment significantly protected these cells against nutrient depletion [17]. The cytoprotective effects of CA were afforded by the phosphorylation of protein kinase B (Akt) and extracellular signal-regulated kinase 1/2 (Erk1/2) and moderate activation of autophagy since pretreatment with LY294002 and U-0126, inhibitors of Akt and Erk1/2 phosphorylation, abolished the protective effects [17]. 

Another mechanism by which CA influences autophagy is through the parkin pathway. Parkin is an E3 ubiquitin ligase that catalyzes the conjugation of ubiquitin to abnormal proteins, facilitating their degradation by the ubiquitin proteasome system (UPS) [18]. Parkin gene mutations have been implicated in the pathogenesis of neurodegenerative diseases, including Parkinson’s [19,20,21]. CA was shown to prevent cell death via induction of the parkin pathway, enhancing levels of parkin protein, the UPS, and α-synuclein degradation [22]. The interaction between parkin and Beclin1 is considered to facilitate autophagosome maturation [23]. CA substantially enhances the parkin/Beclin1 interaction, inducing autophagy [24]. These effects were attenuated by wortmannin and bafilomycin A1 (an autophagosome-lysosome fusion blocker) [24]. Moreover, CA has also been shown to mitigate mitochondrial impairment, which also involves the activation of the PINK1/parkin/mitophagy pathway [25]. The neuroprotective effects of CA have also been attributed to the upregulation of OPA1 (OPA1 mitochondrial dynamin-like GTPase) via activation of the parkin/IKKγ/p65 pathway and are associated with an enhancement of mitochondrial biogenesis. This pathway is linked to the inhibition of Parkin-interacting substrate (PARIS) and induction of proliferator-activated receptor gamma coactivator-1-alpha (PGC-1α) by parkin [26,27]. This interaction has been shown to prevent the degeneration of dopaminergic neurons, demonstrating the therapeutic potential of CA against PD [27]. 

### 2.2. Alleviation of Oxidative Stress

Oxidative stress is a major contributing factor to neurodegenerative disorders [28]. Many studies have highlighted the anti-inflammatory and anti-oxidative properties of CA. Hou and colleagues [29] demonstrated the neuroprotective effect of CA on neuronal cells subjected to ischemia/hypoxia injury via the scavenging or reduction of ROS (reactive oxygen species) and NO (nitric oxide) and inhibition of COX-2 and MAPK pathways [29]. CA also displayed protective effects against 6-hydroxydopamine (6-OHDA)-induced neurotoxicity by increasing the expression of antioxidant enzymes, including c-glutamate-cysteine ligase catalytic (GCLC) subunit, c-glutamate-cysteine ligase modifier (GCLM) subunit, superoxide dismutase (SOD), and glutathione reductase [30]. Furthermore, CA was also demonstrated to be cytoprotective against chlorpyrifos (CPF)-induced inflammation, oxidative stress, and neurotoxicity in brain and eye tissues of mice [31] as well as in SH-SY5Y cells [32]. CA protects against oxidative stress by employing various mechanisms, among which the induction of Nrf2-ARE and the activation of PI3K/Akt signaling pathways are the most significant and widely studied. 

#### 2.2.1. Induction of the Nrf2-ARE Response

The pleiotropic transcription factor, nuclear factor erythroid 2-related factor 2 (Nrf2), is a master regulator of numerous cytoprotective genes. As an important mediator of the endogenous defense system, it serves to combat the imbalance between basal and injury-induced changes in ROS/RNS (reactive nitrogen species) and antioxidant/defense enzymes through its interaction with enhancer regions known as antioxidant response elements (AREs) of defense genes [33,34,35]. Under normal cellular conditions, Nrf2 remains sequestered in the cytoplasm by the regulator protein Keap1 (Kelch-like ECH-associated protein 1) [36], a component of the Cullin 3 (CUL3)-based E3 ubiquitin ligase complex. However, under conditions of cellular stress such as injury, toxicity, or oxidative stress, Nrf2 becomes uncoupled from Keap1 and translocates into the nucleus, where it induces the transcription of cytoprotective genes by binding with AREs [37,38].

**Figure 3:**
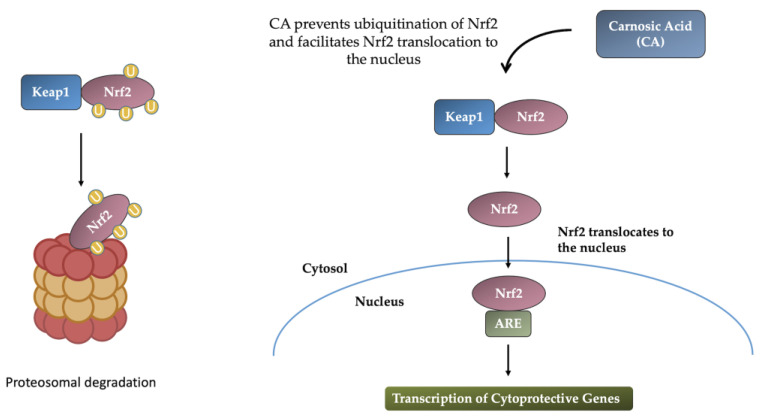
Induction of Nrf2-ARE response by CA. Under physiological conditions, Nrf2 binds to Keap1 and remains sequestered in the cytoplasm, where Nrf2 becomes ubiquitinated, released from Keap1, and degraded by the ubiquitin-proteosome complex. Carnosic acid blocks the ubiquitination of Nrf2 and facilitates its dissociation from Keap1, resulting in its translocation to the nucleus, where it binds to antioxidant response elements (AREs) of cytoprotective genes and facilitates transcription.

As indicated above, CA is known to activate the Keap1/Nrf2 pathway, thereby resulting in the production of cytoprotective proteins. This highlights the significance of CA as a candidate neuroprotective agent for the treatment of neurodegenerative diseases. Activation of the neuroprotective Keap1/Nrf2 transcriptional pathway by CA involves the conversion of CA from an electrophilic precursor to an electrophilic form through a mechanism involving the release of Nrf2 from the Keap1/Nrf2 complex that results in the transcription of antioxidant enzymes (Figure 3) that protect neurons from oxidative stress and excitotoxicity [39]. It has also been shown that the hydrophilicity of CA is critical for its neuroprotective effects, which require both free carboxylic acid and catechol hydroxyl moieties [40]. The mechanism of neuroprotection of CA involves a sequence of events whereby the activation of the Keap1/Nrf2 pathway is followed by the transcription and induction of enzymes involved in glutathione (GSH) metabolism (glutathione S-transferase, alpha 4; glutathione S-transferase, alpha 2; and formylglutathione hydrolase) and phase 2 enzymes [NAD(P)H-quinone oxidoreductase1 and aldehyde dehydrogenase family 3, subfamily A1] that would lead to the activation of GSH metabolism [41]. GSH is a potent antioxidant that protects cells from the toxic effect of ROS.

Interestingly, it has been reported that nerve growth factor (NGF), a proteinaceous neurotrophic molecule essential for the growth and functional maintenance of nervous system tissue, was markedly enhanced in T98G human glioblastoma cells following treatment with CA [42]. Investigations into the mechanisms by which CA increased the production of NGF revealed an Nrf2-dependent pathway whereby treatment increased nuclear accumulation of Nrf2 and the activation of Nrf2 target genes, including heme oxygenase 1 (HO-1) and thioredoxin reductase 1 (TXNRD1) [43]. The neuroprotective mechanism of CA was further delineated in a more recent study demonstrating a CA-mediated induction of the activating transcription factor 4 (ATF4) through the integrated stress response (ISR) pathway. This activation of Nrf2 and ATF4 by CA led to enhanced expression of NGF and other antioxidant genes, including HO-1 and TXNRD1 [44]. CA also worked synergistically with edavarone, a free radical scavenger, to enhance NGF expression in cultured human astrocytes exposed to hypoxia/re-oxygenation [45]. Application of CA to SH-SY5Y cells pretreated with the neurotoxin 6-OHDA facilitated the downregulation of the pro-apoptotic JNK and p38 signaling pathways. This down-regulation was driven by the Nrf2-mediated synthesis of GSH [46]. Similarly, by attenuating the 4-hydroxy-2-nonenal (4-HNE)-induced inhibition of mitochondrial respiration, CA can also alleviate mitochondrial dysfunction. 4-HNE is a by-product of lipid-peroxidation-induced membrane damage and plays a critical role in neurodegeneration. The attenuation of 4-HNE by CA is also associated with the induction of Nrf2-ARE [47]. CA exerts a similar neuroprotective effect through activation of Nrf2-ARE following traumatic-brain-injury-induced oxidative damage and mitochondrial dysfunction [48].

Additional evidence of the neuroprotective effects of CA was demonstrated in cultured rodent and human induced pluripotent stem cell-derived neurons treated with cyanide as well as in a non-Swiss albino mouse model of cyanide poisoning [49]. Acute exposure to cyanide in humans results in a delayed onset (up to weeks or even months) of a neurological syndrome that includes dystonia and signs and symptoms of Parkinsonism. Pretreatment of mice with 0.05% CA in food pellets for 1 week followed by twice daily intraperitoneal administration of 5–6 mg/kg potassium cyanide (KCN) for 8 days whilst maintaining oral treatment (via food) resulted in reduced neurotoxicity and improved neurobehavioral outcomes in treated mice [49]. Importantly, treatment with CA resulted in significantly reduced apoptosis in the frontal cortex, hippocampus, and striatum of KCN-poisoned mice [49]. CA was also capable of differentiating PC12 cells by activating Erk1/2 via the trkA, nerve growth factor receptor, independently of Nrf2 [50]. In addition, CA also affords neuroprotective effects by inhibiting ferroptosis via activation of the Nrf2 pathway [51]. Treatment of PC12 cells with erastin, a ferroptosis inducer, led to a dose-dependent loss in cell viability and decreased glutathione levels that were reversed following treatment with CA. In addition, CA also reversed the reduction in glutathione levels as well as the increase in reactive oxygen and nitrogen species induced by erastin [51].

A study in ovariectomized mice further demonstrated the neuroprotective role of CA in alleviating consequent depressive behaviors through the induction of serotonin and activation of Nrf2/HO-1 signaling [52]. CA also reversed the ovariectomy-induced suppression of the oxidoreductase protein, thioredoxin (Trx-1), and brain-derived neurotrophic factor (BDNF), a pivotal neurotrophic factor associated with neuronal survival. Treatment with CA for three weeks following ovariectomy also suppressed the oxidative stress markers GSH, malondialdehyde, and SOD as well as the pro-inflammatory cytokines TNF-α and IL-1β and ameliorated histopathological changes induced by ovariectomy [52]. Other evidence of the mood-altering effects of CA has been reported [53,54]. Observations of increased serotonin and BDNF suggest that CA may represent a novel therapeutic avenue for depressive behaviors that should be further explored.

#### 2.2.2. Activation of the PI3K/Akt Signaling Pathway

The phosphoinositide-3-kinase (PI3K)/Akt signaling pathway is complex and is involved in numerous cellular functions, including cell growth, metabolism, proliferation, and survival, amongst others. These myriads of functions are driven by the ability of the pathway to regulate a broad spectrum of proteins, including NF-κB (nuclear factor kappa-light-chain-enhancer of activated B cells), another signaling molecule that is predominantly involved in cell survival, inflammation, and protection from toxicity. CA has been shown to mediate the activation of the PI3K/Akt/NF-κB pathway, leading to the upregulation of GSTP (Glutathione S-transferase pi) [55], one of the seven classes of GSTs and one that is highly expressed in glial cells of the nervous system. Upregulation of GSTP enzyme activity was shown to attenuate 6-OHDA-induced apoptosis and cell death both in SH-SY5Y neuroblastoma cells as well as in the striatum of mice [55]. Similarly, employing methylglyoxal (MG), the most potent inducer of advanced glycation end-products (AGEs), de Oliveira and colleagues [56] demonstrated that pretreatment of SH-SY5Y cells with CA prevented cells from damage caused by free radicals produced during the metabolism of MG. The cytoprotective effects of CA were exerted via activation of the PI3K/Akt/Nrf2 signaling pathway, where antioxidant enzymes were modulated by Nrf2 [56]. CA prevented MG-induced cell death by increasing levels of Bcl-2 (an apoptosis regulating protein) and decreasing levels of Bax (another apoptosis regulator), as well as by blocking cytochrome c release from mitochondria and the loss of mitochondrial membrane potential (MMP) induced by MG. CA pretreatment also inhibited caspase-3 and caspase-9 activation and decreased the fragmentation of DNA that is generally elicited by MG [56]. Similar effects of CA were observed employing the paraquat (PQ) model of PD, where cytoprotection was afforded by activation of Nrf2 through modulation of the PI3K/Akt pathway leading to an increase in levels of antioxidant enzymes [32,56,57]. It is further suggested that CA also exerts mitochondrial protection from glutamate-induced excitotoxicity. Results in SH-SY5Y cells treated with CA revealed prevention of glutamate-induced mitochondrial impairment and improved bioenergetics that was driven through the activation of Nrf2 [58].

### 2.3. Attenuation of Apoptosis

Although many studies highlight the role of CA in modulating autophagy, as discussed earlier, it is also found to play a critical role in the attenuation of apoptosis. Investigations have used variously in vitro and in vivo models of apoptosis to evaluate the neuroprotective role of CA and have revealed regulation at the level of apoptosis-inducible genes [59]. Studies in cultured dopaminergic cells (SN4741) employing the organochlorine pesticide dieldrin, which is known to be a risk factor for PD, revealed that neuroprotection afforded by CA was due to the repression of apoptosis-related caspase-3 and -12 and the stress signaling molecule c-Jun N-terminal kinase (JNK) [60]. Pretreatment of SN4741 cells with CA also significantly attenuated the downregulation of BDNF, a key molecule associated with dopaminergic neuron survival and maturation [60]. Treatment of these cells with dieldrin resulted in a 61% reduction in BDNF release from these cells, whereas pretreatment with 10 μM CA maintained levels of BDNF at basal expression [60]. Intriguingly, these results suggest that treatment of SN4741 cells with 10 μM CA results in a 1.5-fold increase in levels of BDNF, suggesting that prophylactic treatment with CA may support dopaminergic and other cells in the brain. In another example of the neuroprotective effects of CA, Wu and colleagues [30] reported that the cytoprotective effects of this diterpenoid were afforded by its anti-apoptotic action in 6-OHDA-treated rats and SH-SY5Y cells. This effect was mediated by Bax, a pro-apoptotic, and Bcl-2, an anti-apoptotic member of the Bcl-2 family of proteins. Treatment with CA was shown to reverse the 6-OHDA-induced reduction in the Bcl-2/Bax ratio [30]. CA also decreased 6-OHDA-induced apoptosis in SH-SY5Y cells via upregulation of GSTP through the activation of the PKA/CREB pathway and subsequent increase in the interaction between GSTP and JNK, resulting in an inhibition of JNK signaling [61].

Another study investigating the mechanism by which CA inhibits apoptosis revealed the role played by the E3 ubiquitin ligase, parkin. As indicated, parkin ubiquitinates misfolded proteins and facilitates their degradation via the ubiquitin-proteasome system [62,63]. Treatment of SH-SY5Y cells with 6-OHDA induced the expression of apoptosis-related protein in the TGF-β signaling pathway (ARTS), a pro-apoptotic protein, and reduced the expression of X-liked inhibitor of apoptosis protein (XIAP), a protein that directly blocks active sites of caspase 3 and caspase 7 and inhibits apoptosis. Pretreatment of SH-SY5Y cells with CA ameliorated the induction of ARTS and reduction of XIAP and also attenuated the activation of caspase 7 and 9, thereby reversing the apoptotic effects of 6-OHDA and shedding light on the therapeutic potential of CA in PD [64].

CA was also reported to exert a neuroprotective effect following subarachnoid hemorrhage induced by early brain injury through the inhibition of apoptosis [65]. Rats were subjected to a sub-arachnoid hemorrhage procedure, and those in the experimental group were then administered a 3 mg/kg dose of CA intraperitoneally. CA was shown to ameliorate brain edema and blood-brain barrier (BBB) disruption, as well as reduce neuronal death via apoptosis [65]. CA was also shown to increase SIRT1, a member of the highly conserved (NAD+)-dependent class of histone deacetylases responsible for combatting ROS and apoptosis, MnSOD (manganese superoxide dismutase, a metalloprotein that prevents mitochondrial dysfunction) and Bcl-2 (the founding member of a family of regulator proteins that regulate cell death) expression [65], as well as decreased p66shc, Bax, and cleaved caspase-3 expression. The anti-apoptotic effects of CA were proposed to be facilitated through the SIRT1/p66shc signaling pathway [65,66]. 

Importantly, CA was shown to inhibit cell growth and induce apoptosis in IMR-32 human neuroblastoma IMR-32 cells [67]. The induction of apoptosis was accompanied by ROS-mediated p38 MAPK activation resulting in a decrease in cell viability [67]. Intriguingly, these results suggest that the activity of CA is selective in its regulation of cell viability and apoptosis, whereby these processes are activated by CA to restore physiological states, implying the substantive therapeutic potential of this compound that warrants extensive investigation.

### 2.4. Effects of Carnosic Acid in Amyloid-β-Mediated Neurodegeneration

Brain atrophy associated with the deposition of Aβ in extracellular neuritic plaques is the most prominent neuropathological hallmark of Alzheimer’s disease (AD) [68]. Aβ-peptide, which constitutes the major component of amyloid plaques, is a 4-kDa peptide formed by the proteolytic cleavage of the amyloid precursor protein (APP) by β-secretase and the γ-secretase complex of proteins [69,70]. Cleavage of APP by β-secretase (β-site APP-cleaving enzyme-1 (BACE1)) catalyzes the critical step in the generation of Aβ. However, the constitutive pathway of APP processing is via α-secretase cleavage that results in the generation of a soluble ectodomain fragment termed soluble APPα (sAPPα), which possesses neurotrophic and neuroprotective properties [71,72,73]. The protective role of CA against neurodegeneration resulting from the presence of Aβ is well documented. An investigation of the effects of CA on Aβ production in SH-SY5Y human neuroblastoma cells revealed a critical role for this antioxidant in the suppression of Aβ_42_ generation, an isoform of the peptide that is known to be more hydrophobic and toxic as well as possessing faster oligomerizing properties compared to Aβ_40_. In the presence of CA, APP cleavage was shuttled to the α-secretase pathway, thereby precluding Aβ generation [74]. This shuttling in the presence of CA is driven by the upregulation of tumor necrosis factor-α-converting enzyme (TACE) mRNA, a member of the ADAM (a disintegrin and metalloproteinase) family of proteases, which contributes to α-secretase cleavage of APP [74]. Similarly, a substantial reduction in Aβ production by CA via the activation of TACE was evident in U373MG human astrocytoma cells [75]. Aβ also interacts with N-methyl-D-aspartate receptors (NMDARs) to induce apoptosis and synaptic dysregulation. In another study on SH-SY5Y cells, CA was shown to inhibit the phosphorylation of the NMDAR subtype 2B (NMDAR2B) receptor, thereby suppressing apoptosis and restoring expression of synaptic proteins including BDNF, postsynaptic density protein-95 (PSD-95), and synaptophysin [76]. Additionally, CA significantly attenuated apoptosis induced by Aβ_42/43_, further highlighting its therapeutic potential against Aβ-induced neurotoxicity [77]. 

In vivo, CA has been demonstrated to be protective to neurons in subfield CA1 (cornu Ammonis) of the hippocampus in an acute experimental rat model of AD (bilateral administration of Aβ into the hippocampus) where Aβ accumulation leads to neurodegeneration of the hippocampus [78]. Employing a similar in vivo paradigm, Rasoolijazi and colleagues [79] demonstrated the neuroprotective effects of CA on cognitive impairment associated with Aβ-induced neurotoxicity in the rat hippocampus. CA was shown to significantly improve short-term and spatial memory attributes in rat models of AD [79]. Furthermore, CA also delayed the deposition of Aβ and protected cells against Aβ-induced cholinergic and mitochondrial dysfunction in a Caenorhabditis elegans model of AD [80], thereby reiterating its promising potential as a neuroprotective agent against AD-associated neurodegeneration.

In recent efforts incorporating biomedical advances, nano-carrier packaged CA reduced the deposition of Aβ, subsequently restoring cognitive deficits through the inhibition of the CCAAT-enhancer-binding protein β (CEBPβ)-NFκB signaling pathway in APP/PS1 mice [81].

A recent study by Feng and colleagues [82] demonstrated a potential role of CA in the suppression of Apolipoprotein E ε4 (ApoE ε4)-associated AD. Apolipoprotein E is a major cholesterol transport protein. The ε4 allele of APOE is the strongest risk factor for late-onset AD (LOAD), the most common form of the disease that affects more than 97% of individuals diagnosed with AD. An increase in the cell surface expression of ApoE receptor 2 (ApoER2) activates the reelin signaling pathway that is important for synaptic plasticity in the adult brain. The intracellular binding of ApoE4 to ApoER2 inhibits the recycling of the receptor to the cell membrane and therefore renders neurons unresponsive to reelin [83]. CA counteracts the negative effects of ApoE ε4 by facilitating the binding of sorting nexin 17 (SNX17) to ApoER2, blocking ApoE ε4 binding and promoting the recycling of the receptor to the cell membrane [84] where reelin binds to the receptor, activating the pathway resulting in neurite growth [82]. 

### 2.5. Effects of Carnosic Acid in Models of Neuronal Injury

Intriguingly, CA also alleviated symptoms of metabolic-disease-induced brain injury through the modulation of inflammatory responses. In a high-fat-diet-induced mouse model, CA facilitated a significant decrease in the expression of various pro-inflammatory cytokines regulated by the NF-κB signaling pathway, including interleukin (IL)-1β, IL-6 and tumor necrosis factor-α (TNF-α). Additionally, it also modulated the apoptotic pathway through the increased expression of anti-apoptotic Bcl-2 and downregulation of the pro-apoptotic protein Bax and matrix metallopeptidase 9 (MMP9) [85]. 

Studies in levodopa-induced dyskinesia revealed that CA was capable of alleviating the detrimental effects of excessive levodopa through the attenuation of apoptotic cell death via the modulation of ERK1/2-c-Jun and induction of parkin [86]. It also attenuated inflammation, mitochondrial damage, and oxidative stress in isoflurane-treated neuronal cells through the activation of the AMPK/SIRT1 pathway [87]. CA has also been shown to exert anti-inflammatory responses in bone-marrow-derived macrophages through the modulation of the toll-like receptor 2 (TLR2) and MAPK/NF-κB signaling pathway, resulting in a decreased expression of TNF-α, IL-6, and IL-1β [88]. The anti-inflammatory response of CA was further demonstrated via an integrated proteomic and bioinformatic study that demonstrated the involvement of CA in the modulation of multiple inflammatory processes, including MAPK, NF-κB, and FoxO signaling pathways [89]. CA also inhibits the nucleotide-binding oligomerization domain-like receptor containing pyrin domain 3 (NLRP3) inflammasome, which plays a critical role in the pathogenesis of neurodegenerative disorders, including AD and PD and COVID-19, including ‘long-COVID’, thereby representing its therapeutic potential [90]. Additionally, its neuroprotective role in the prevention of prion protein (PrP) aggregation in cellular models as well as disruption of PrP aggregates in cell-free assays [91], raises interesting possibilities for considering CA as a potential adjuvant candidate against prion diseases, including Creutzfeldt–Jakob disease (CJD), Gerstmann–Straussler–Scheinker disease (GSS), and fatal familial insomnia (FFI).

Collectively, these studies demonstrate the cytoprotective characteristics afforded by CA and support its use as both a prophylactic and a neuroprotective compound that warrants continued investigation in diseases of the nervous system (summarized in Table 1).

## 3. Conclusions

The research discussed above reveals the neuroprotective effects of carnosic acid, the most abundant compound found in plants belonging to the family *Lamiaceae*, including rosemary and sage. When used either as a prophylactic or as a therapeutic, CA is capable of mitigating the damage caused to nervous system tissue, thereby revealing a unique role in the management of neurodegenerative disorders. A deeper understanding of the neuroprotective properties of CA will facilitate the broader applicability of this intriguing compound and may aid in its use in conjunction with mainstay treatments for neurological disorders. 

## Figures and Tables

**Table 1:** Neuroprotective effects of carnosic acid and its associated mechanisms of action.

Neuroprotective Effects	Mechanisms
Induction of autophagy	Activation of AMP-activated protein kinase (AMPK) [16]
	Phosphorylation of protein kinase B (Akt) and extracellular signal-regulated kinase 1/2 (Erk1/2) [17,86]
	Induction of Parkin pathway [22,86]
	Enhancement of parkin/Beclin1 interaction [24]
	Activation of the PINK1/parkin/mitophagy pathway [25]
	Activation of the parkin/IKKγ/p65 pathway [26,27]
Alleviation of oxidative stress	Induction of Nrf2-ARE response [39,40,41,42,43,44,45,46,47,48,49,50,51,52]
	Activation of the PI3K/Akt signaling pathway [32,55,56,57,58]
Attenuation of apoptosis	Repression of apoptosis-related caspase-3 and -12 and c-Jun N-terminal kinase (JNK) [60,61]
	Attenuation of BDNF downregulation [60]
	Restoration of Bcl-2/Bax ratio [30]
	Activation of the PKA/CREB pathway [61]
	Amelioration of the induction of ARTS and reduction of XIAP [64]
	Activation of SIRT1/p66shc signaling pathway [65]
Protection against Aβ-mediated neurodegeneration	Upregulation of tumor necrosis factor-α-converting enzyme (TACE) mRNA to suppress Aβ_42_ generation [74,75]
	Inhibition of NMDAR subtype 2B (NMDAR2B) receptor phosphorylation [76]
	Restoration of cognitive impairment [78,79]
	Suppression of Aβ-induced cholinergic and mitochondrial dysfunction [80]
	Inhibition of the CCAAT-enhancer-binding protein β (CEBPβ)-NFκB signaling pathway [81]
	Suppression of Apolipoprotein E e4 (ApoE e4)-associated AD [82]
Protective role in models of neuronal injury	Suppression of various pro-inflammatory cytokines [85]
	Activation of AMPK/SIRT1 pathway [87]
	Modulation of the toll-like receptor 2 (TLR2), MAPK/NF-κB, and FoxO signaling pathway [88,89]
	Inhibition of the nucleotide-binding oligomerization domain-like receptor containing pyrin domain 3 (NLRP3) inflammasome [90]
	Prevention of prion protein (PrP) aggregation [91]

## Data Availability

The data presented in this study are available in the article.
